# Current status of post-traumatic stress disorder among emergency nurses and the influencing factors

**DOI:** 10.3389/fpsyt.2023.1203782

**Published:** 2023-09-04

**Authors:** Yu-Fei Qian, Ying Liu, Li Wang, Qing Li, Rong-Qian Sun

**Affiliations:** ^1^Department of Emergency, The Second Affiliated Hospital of Nantong University, Nantong, China; ^2^Department of Nursing, The Second Affiliated Hospital of Nantong University, Nantong, China

**Keywords:** emergency nurses, influencing factors, post-traumatic stress, traumatic events, current status

## Abstract

**Objective:**

To gain a better understanding of the current state of traumatic stress experienced by emergency nurses of Grade III Level A hospitals in Jiangsu Province, as well as their coping styles after experiencing such traumatic events. Additionally, this study aims to identify the primary factors that influence the responses of these nurses to traumatic events.

**Methods:**

Using a cluster random sampling method, we enrolled 265 nurses working in the emergency departments of five Grade III Level A hospitals in Jiangsu Province. These nurses participated in a questionnaire survey that included inquiries regarding general information, previous traumatic experiences, and a post-traumatic stress disorder self-assessment scale (PCL-C) for emergency department nurses.

**Results:**

A total of 290 questionnaires were distributed, resulting in 265 valid questionnaires and an effective rate of 91.38%. These findings indicated that emergency nurses who participated in public health emergencies such as the COVID-19 pandemic (45.66%) and sudden health deterioration and death of patients (43.77%) encountered the most traumatic events. The top two traumatic events that had a moderate or greater impact on emergency nurses were verbal abuse from patients or their family members (39.24%) and verbal or physical threats by patients or their family members (35.09%). The mean PCL-C score of nurses who experienced traumatic events was 33.62 ± 11.37, with a positive rate of 26.04%. Results from the one-way analysis of variance and multiple linear regression analysis demonstrated that the working years, monthly income, and personal health status of emergency nurses were the main factors contributing to post-traumatic stress disorder.

**Conclusion:**

Emergency nurses are susceptible to severe traumatic stress following traumatic events, and effective interventions are necessary to address the diverse factors that contribute to their psychological well-being.

## 1. Introduction

Post-traumatic stress disorder (PTSD) has become a significant topic of clinical research in recent years, with various groups, including patients, doctors, and nurses as research subjects. In recent years, studies outside of China have reported that the prevalence of PTSD among nurses across different hospital departments ranges from 29 to 42.4% (1–3); whereas the rate is even higher among emergency department nurses (4, 5), with a prevalence ranging from 43.9 to 57.2%. In China, Qiong et al. conducted a survey on 103 nurses in pre-hospital emergency care, revealing a high prevalence rate of 34.0% (6). The impact of PTSD extends beyond the individual’s personal life, as it can lead to decreased motivation, job dissatisfaction, and even a strong desire to leave one’s job and profession (7).

Traumatic events such as child deaths, serious accidental injuries suffered by workers, and public health emergencies are known to be the direct cause of PTSD among emergency health care workers. With the outbreak of the COVID-19 pandemic in 2019, the prevalence rate of PTSD has increased significantly among health care workers who have been working tirelessly on the front line to prevent and control the pandemic (8). Surveys conducted by Cai et al. during the peak and stable phases of the pandemic have shown that the positive rate of PTSD was significantly higher among nurses during the peak period than during the stable period (9). Another investigation revealed that even 2 years after the Wenchuan earthquake in 2008, the positive rate of PTSD among those participating in earthquake relief was still 1.7% (10). This shows that sudden public health events and catastrophic events are significant causes of PTSD among health care workers alongside traumatic events.

Previous studies have demonstrated that nurses may experience persistent PTSD even years after traumatic events. Failure to address the psychological well-being of nurses with PTSD in a timely manner may result in higher error rates in nursing, increased medical and nursing risks, reduced patient satisfaction, and a negative impact on the overall medical and nursing environment, leading to serious consequences for both the nurses’ physical and mental health as well as their families. Thus, it is of great importance to better understand the prevalence of PTSD among nurses and implement early intervention strategies. This study aimed to investigate the occurrence and impact of traumatic events experienced by emergency nurses in five tertiary general hospitals in Jiangsu Province, China, through a comprehensive questionnaire survey. By understanding the current status of emergency traumatic events in the Chinese social environment and their impact, and assessing the psychological state of emergency nurses, including the presence of PTSD and relevant influencing factors, we aimed to provide targeted prevention and intervention strategies. This approach is crucial for protecting the mental health of nurses, reducing nursing-related risks, and maintaining a positive medical care environment.

## 2. Participants and methods

### 2.1. Study participants

From March to May 2022, a cluster random sampling method was used to enroll nurses working in the emergency departments of five Grade III Level A hospitals in Jiangsu Province for a questionnaire survey. Inclusion criteria: (1) nurses licensed and registered as a nurse practitioner, (2) non-retraining nurses, and (3) nurses who volunteered to participate in this study. Exclusion criteria: (1) those not on duty during the survey, (2) those who were pregnant or in the lactation period, (3) those who had experienced serious life incidents within the last year or during the survey such as serious illness or death of family members, and (4) those with a history of anxiety disorders, depression, and other psychiatric disorders. The study was approved by the Ethics Committee of Nantong First People’s Hospital, and trained staff provided instructions to the study participants on how to fill out the form. No leading language was allowed during the survey to ensure the accuracy of the responses.

### 2.2. Survey tools

#### 2.2.1. General information of questionnaire

The general information includes gender, age, education level, marital status, title, years of work in emergency nursing, and other aspects.

#### 2.2.2. Questionnaire of traumatic events for emergency nurses

The *Traumatic Events Questionnaire for Nurses in Emergency Departments* (hereinafter referred to as the Event Questionnaire), compiled by Lin (11), was used in this study. It consists of five sections, namely death stimuli (6 items), treatment and cure of special patients (6 items), workplace violence (4 items), negative work-related events (3 items), and public health emergencies (4 items). The questionnaire has good reliability and validity, with a test–retest reliability of 0.718, the coefficient Cronbach’s ɑ of 0.835, and a CVI value of 0.845. The event questionnaire was scored based on the psychological impact of traumatic events, with a rating scale of 1 to 5. A score of 1 point indicates “no influence,” 2 points indicates “mild,” 3 indicates “moderate,” 4 points indicates “severe,” 5 points indicates “very severe.” If no traumatic event occurs, the score was classified as “no influence.”

#### 2.2.3. PTSD checklist-civilian version (PCL-C)

The *PTSD Checklist-Civilian Version* (PCL-C) was compiled by Weathers et al. in 1991 (12), and the revised PCL-C consists of 17 items that are closely related to the diagnosis of PTSD in the DSM-IV criteria. The questionnaire assesses three key aspects of PTSD symptoms: repeated reoccurrences (5 items), avoidance symptoms (7 items), and increased alertness (5 items). Participants were asked to rate each item on the Likert 5-point scale, with 1 indicates “not at all,” 2 indicates “a little,” 3 indicates “moderate,” 4 indicates “considerable,” and 5 indicates “extreme.” The total PCL-C score ranges from 17 to 85, with a total score of 38 and higher indicating a positive diagnosis of PTSD. A higher total score indicates a higher degree of stress disorder.

### 2.3. Sample size calculation

Sample size calculation for quantitative research follows the guideline of having 5–10 times the number of variables. Considering a preset questionnaire failure rate of 10–20%, this study used the “Emergency Department Nurses Traumatic Incident Questionnaire” and the “Post-Traumatic Stress Disorder Self-Assessment Scale (PTSD Checklist-Civilian Version, PCL-C)” with a total of 40 variables. Based on this, the estimated sample size ranged approximately between 220 and 480 cases.

### 2.4. Collection method of data

We used Sojump to compile the questionnaires, which were then dispatched via various channels such as WeChat and email. The head nurses of the emergency department of the hospitals where the respondents worked were responsible for distributing the questionnaires. To ensure the accuracy and authenticity, the head nurses supervised the completion of the questionnaires. Any completion time exceeding 8 min was considered valid, which was supervised by the head nurses.

### 2.5. Statistical methods

We performed statistical analysis using SPSS 26.0 software. Descriptive statistics were used for general demographic data, with count data presented as composition ratios, frequencies, and percentages. Measurement data were expressed as mean ± standard deviation and compared by ANOVA. To identify factors that contribute to post-traumatic stress disorder in emergency nurses, we conducted both one-way analysis and multiple regression analysis. Statistical significance was considered at *p* < 0.05.

## 3. Results

### 3.1. General situation

We conducted a survey of 290 nurses working in emergency departments of five Grade III Level A hospitals in Jiangsu Province. We received 265 valid questionnaires, resulting in an effective rate of 91.38%. Of the 265 participants, 41 (15.47%) were male and 224 (84.53%) were female. In terms of job positions, 66 (24.91%) were nurses, 112 (42.26%) were senior nurses, and 87 (32.83%) were supervisor nurses or higher. Details of the information on demographic characteristics are shown in Table 1.

**Table 1:** Information on different demographic characteristics of emergency nurses (*n* = 265).

Item		Number of cases	Percentage (%)
Gender	Male	41	15.47
	Female	224	84.53
Age (yrs)	18 ~ 29	131	49.43
	30 ~ 44	123	46.42
	Over 45 years old	11	4.15
Marital status	Unmarried	110	41.51
	Married	148	55.85
	Divorced or other	7	2.64
Education level	Junior college and below	53	20
	Bachelor’s degree and above	212	80
Title	Nurse	66	24.91
	Senior nurse	112	42.26
	Supervisor nurse and above	87	32.83
Years of working in emergency nursing	Less than 1 year	27	10.19
	1 to 3 years	48	18.11
	3 to 5 years	56	21.13
	5 to 10 years	65	24.53
	10 years or more	69	26.04
Personal monthly income	2000 ~ 4,000	26	9.81
	4,001 ~ 6,000	60	22.64
	6,001 ~ 8,000	68	25.66
	More than RMB 8,001	111	41.89
Your current health condition	Normal	133	50.19
	Fair	124	46.79
	Poor	8	3.02

### 3.2. Investigation of traumatic events among emergency nurses

According to the survey, the top three traumatic events that emergency nurses frequently face are participation in public health emergencies including the fight against the new coronavirus epidemic (45.66%), sudden deterioration of patient condition and death (43.77%), and verbal abuse by patients or their family members (44.53%). Moreover, the top three traumatic events that had more than moderate impact on emergency nurses are verbal abuse by patients or their family members (39.24%), verbal or physical threats by patients or their family members (35.09%), sudden death of young adults (29.81%), and child death (29.81%). Please refer to Table 2 for details.

**Table 2:** Traumatic event survey for nurses in emergency department (%).

Item	Did not occur	Frequently faced	Within 3 months	Within 6 months	Within 1 year	1 year ago	No mental impact	Mild impact	Moderate impact	Severe impact	Very severe effects
Sudden deterioration of patient condition and death	16.23	43.77	14.34	6.79	9.06	9.81	38.11	47.18	12.83	1.13	0.75
Patient forgoes treatment due to cost and dies	22.64	38.49	16.98	6.04	10.94	4.91	42.26	41.14	12.45	3.4	0.75
Patient commits suicide	44.53	23.02	10.94	6.42	8.3	6.79	55.09	31.32	8.68	3.4	1.51
Sudden death of young adults	24.53	24.91	15.85	9.05	19.25	6.41	38.49	31.7	20.75	6.42	2.64
Child death	44.15	11.32	7.92	7.18	15.09	14.34	47.92	22.27	16.98	8.3	4.53
Care for a corpse	32.83	40	9.43	3.4	7.55	6.79	55.47	28.3	10.57	4.53	1.13
Care for patients with severe burns or trauma	30.57	31.32	10.94	7.93	11.32	7.92	50.19	30.95	12.83	5.28	0.75
Care for patients with mental disorders and violent behavior	34.34	20	12.83	8.3	13.96	10.57	52.45	32.08	11.7	3.02	0.75
Care for patients with delirium	23.02	42.26	12.45	6.42	9.81	6.04	54.34	33.21	10.19	1.88	0.38
Care for patients who committed suicide	21.51	34.34	18.11	6.79	10.19	9.06	52.45	34.34	11.32	1.51	0.38
Care for sick relatives and friends	56.98	10.57	5.66	6.04	7.92	12.83	53.21	20	14.34	7.17	5.28
Verbal abuse by patients or their family members	19.25	44.53	15.47	4.53	10.94	5.28	31.7	29.06	25.66	9.43	4.15
Verbal or physical threats by patient or their family members	27.92	32.08	13.96	4.15	13.97	7.92	38.49	26.42	22.26	9.43	3.4
Physical assault by patient or their family members	57.74	16.6	6.04	3.77	7.17	8.68	52.45	20.38	16.98	6.79	3.4
Sexual harassment	86.80	1.13	1.13	2.26	5.66	3.02	71.7	9.43	9.05	4.91	4.91
Participate in the fight against the Covid-19 epidemic	20	45.67	11.32	7.92	9.81	5.28	49.81	30.19	14.34	4.53	1.13
Participate in providing first aid during a major disaster	62.26	10.19	5.28	3.4	8.68	10.19	64.53	22.64	9.82	2.26	0.75
Treat many patients with food poisoning at one time	43.4	11.32	8.68	3.77	15.85	16.98	63.4	26.79	7.17	2.26	0.38
Care for many trauma patients at one time	42.26	15.47	12.08	4.53	12.08	13.58	58.87	24.91	12.45	2.64	1.13
Care for many patients with gas poisoning	47.56	10.19	10.94	7.92	9.81	13.58	64.15	23.4	9.81	1.89	0.75

### 3.3. Levels of post-traumatic stress disorder among emergency nurses

**Figure 1:**
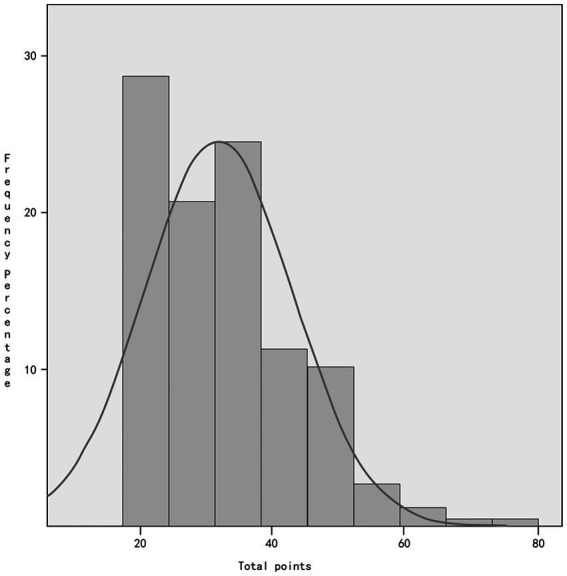
Frequency distribution of total PCL-C scores of emergency nurses.

The survey results indicated that among the 265 emergency nurses who participated, their PCL-C scores ranged from 17 to 74, with a mean score of 33.62 ± 11.367. Of the total, 69 emergency nurses scored 38 or more, indicating a positive rate of 26.04%. The mean score for repeated traumatic events was 11.26 ± 3.59 points, while the mean scores for avoidance response and increased alertness were 12.26 ± 4.79 points and 10.1 ± 3.94 points, respectively. Refer to Figure 1 for more details.

### 3.4. Analysis of factors influencing PTSD symptoms in emergency nurses

Emergency nurses were grouped based on their different demographic characteristics, and their PCL-C scores were used as the dependent variable. The results of univariate analysis revealed that there were statistically significant differences (*p* < 0.05) in three aspects—emergency nurse seniority, monthly income, and health status, as detailed in Table 3. The three factors of seniority, monthly income, and health status were used as independent variables into regression equation, which yielded *R^2^* of 0.224, an adjusted *R^2^* of 0.215, an *F* value of 25.172, and a *p* value less than 0.05. The regression equation was statistically significant, as detailed in Table 4.

**Table 3:** Univariate analysis of PTSD levels by demographic characteristics (*n* = 265).

Item		Number of cases	PCL-C scores	F	*p*
Gender	Male	41	31.85 ± 6.65	0.978	0.517
	Female	224	31.58 ± 3.74		
Age (yrs)	18 ~ 29	131	30.28 ± 6.32	1.120	0.294
	30 ~ 44	123	33.34 ± 7.91		
	Over 45 years old	11	28.36 ± 2.16		
Marital status	Unmarried	110	30.51 ± 5.74	1.158	0.246
	Married	148	32.76 ± 6.85		
	Divorced or other	7	25.14 ± 3.77		
Education level	Junior college and below	53	40.00 ± 1.64	0.597	0.979
	Bachelor’s degree and above	212	32.43 ± 3.79		
Title	Nurse	66	26.89 ± 3.11	1.345	0.078
	Senior nurse	112	32.69 ± 8.04		
	Supervisor nurse and above	87	33.84 ± 6.30		
Years of working in emergency nursing	Less than 1 year	27	23.19 ± 4.18	1.703	0.007
	1 to 3 years	48	29.08 ± 3.76		
	3 to 5 years	56	33.82 ± 5.21		
	5 to 10 years	65	33.86 ± 1.38		
	10 years or more	69	32.80 ± 4.22		
Personal monthly income	2000 ~ 4,000	26	25.12 ± 3.34	1.577	0.018
	4,001 ~ 6,000	60	30.42 ± 6.70		
	6,001 ~ 8,000	68	29.35 ± 2.01		
	More than RMB 8,001	111	35.19 ± 2.28		
Your current health condition	Normal	133	27.50 ± 6.17	2.388	0.000
	Fair	124	34.73 ± 3.52		
	Poor	8	52.13 ± 4.38		

**Table 4:** Multiple linear regression analysis of factors influencing PTSD occurrence among emergency nurses.

Variables	Non-standardized coefficient		Standardized coefficient *β*	*t*	*p*
	*β*	SE			
Constant term	12.104	2.452		4.936	0.000
Years of working in emergency nursing	0.342	0.581	0.040	0.588	0.557
Personal monthly income	2.054	0.743	0.184	2.765	0.006
Current health status	7.989	1.135	0.392	7.039	0.000

## 4. Discussion

### 4.1. Current status of traumatic events among emergency nurses

Traumatic events are a significant contributor to PTSD among emergency nurses, and the survey showed that almost all emergency nurses had experienced at least two traumatic events in their line of work. The event that occurred most frequently was the “sudden deterioration of patient condition and subsequent death.” In terms of scoring, workplace violence was rated the highest and had the most substantial impact, followed by death stimuli and public health emergencies. According to the survey, the most common traumatic event faced by emergency nurses is the “sudden deterioration of patient condition and death.” Although this event occurs frequently due to the special nature of emergency work, emergency nurses may have a better understanding of this high frequency event, and its psychological impact may not be as severe. Workplace violence (WPV) is a significant public safety issue in the global healthcare industry, and is classified into physical and psychological violence based on its type (13). Emergency departments have a high prevalence of WPV, and approximately 90% of emergency nurses have experienced workplace violence (14). According to this survey, a mere 19.25% of the workers in emergency departments in the surveyed area had not experienced “verbal abuse by patients or their family members.” Shockingly, as many as 42.26% of emergency nurses had experienced “physical assault by patients or their family members,” with “workplace violence” emerging as the primary factor affecting the mental well-being of emergency nurses. In a semi-structured review of emergency personnel in Sweden regarding WPV (15), it was observed that experiencing WPV can negatively affect the psychological state of emergency personnel, leading them to adopt resistance and avoidance strategies to cope with violent incidents. If not addressed on time, this could result in personal, professional, and occupational changes. WPV has had a severe impact on the mental health of emergency personnel. In public health emergencies, emergency nurses may face higher work stress and a higher prevalence of PTSD compared to nurses in other departments (16, 17), which aligns with the findings of this study. The outbreak of the novel coronavirus pneumonia epidemic (Covid-19) is the most serious global public health emergency in the past 4 years. Medical workers at the forefront of emergency relief have been under immense physical and mental stress during the treatment, prevention, and control of the epidemic, and face a significantly high risk of PTSD (18). Since the outbreak of the Covid-19 epidemic in early 2020, healthcare medical workers across the country have been participating in the fight against the epidemic, and the ongoing prevention and control of the Covid-19 epidemic has left frontline emergency medical and nursing staff in a state of chronic fatigue. As per our study results, 80% of emergency nurses participated in the epidemic prevention and control efforts, and over 50% of them have been affected psychologically. In December 2022, the adjustment of the Chinese epidemic prevention and control policy and short-lived peak of infection led to a significant shortage of nursing staff., It remains to be seen whether this impact will cause a new peak of PTSD among emergency nurses and what the post-traumatic stress level of emergency nurses is. Further research is required to investigate these questions.

### 4.2. Analysis of the occurrence of PTSD symptoms of emergency nurses

PTSD is currently a significant factor that affects the physical and mental health of medical workers and has garnered widespread attention globally. In this study, we discovered that 265 emergency department nurses had scores ranging from 17 to 74, with a mean score of 33.62 ± 11.367 and a high positive rate of 26.04%. These results are similar to those of some recent worldwide studies (5, 19, 20). It may be due to the fact that emergency nurses are faced with more stressors, as well as a higher workload and work pressure. A study conducted in Iran demonstrated the risk of PTSD was much higher in frontline emergency workers than in non-frontline personnel after the Covid-19 epidemic (21). The results of our study also demonstrated an increasing trend in the level of PTSD among emergency nurses in China during the same period. The results of the two studies are consistent, which may be related to the prolonged duration of the Covid-19 epidemic and the high intensity of work. This suggests that continued long-term response to public health emergencies may lead to a high positive rate of PTSD among emergency nurses. Similarly, it has been shown that after experiencing a public health emergency or catastrophic event, those who experience more traumatic events and the more severe the nature of the event have higher levels of PTSD (22), which is consistent with the results of our study.

### 4.3. Analysis of factors influencing the occurrence of PTSD among emergency nurses

The results of the univariate analysis revealed that the main influencing factors for the occurrence of PTSD among emergency nurses were their seniority, monthly income, and personal health status. Surprisingly, gender, title, age, and education background did not significantly influence the occurrence of PTSD, contrary to common perceptions (23, 24). This phenomenon may be due to the continuous development of nursing teams, and the increasing maturity of emergency nursing teams, which enables less experienced nurses to compensate for anxiety and lack of expertise through teamwork. However, in a study conducted by Niu et al. in the Ningxia region, it was found that female health care workers had a higher rate of positive PTSD in the presence of public health emergencies than male workers (25), which is inconsistent with the results of this study. This discrepancy may be due to regional differences in care patterns, which highlights the need for further studies on the level of PTSD among emergency nurses nationwide. The results of our study revealed that the longer the time spent in the emergency department, the higher the degree of PTSD the nurses experienced. This could be attributed to the negative psychological impact of the fast-paced, high-intensity work in the emergency department becoming worse over time. The exhaustion caused by continuous high-intensity work cannot be alleviated well, which exacerbates the situation. This finding is in line with the study by Peterson et al. (17) Additionally, the results of our study revealed that salary is also an important factor leading to PTSD among emergency nurses, particularly after experiencing public health emergencies such as the Covid-19 epidemic. Job security and income are common concerns and important stressors for health care workers, which is consistent with the findings of James et al. (26) Social support is an important resource. Scholars in Japan have suggested that a positive correlation exists between the social support system and the level of PTSD among healthcare workers (27). This demonstrates that improving the job security system can instill a sense of value in healthcare workers that matches their occupational risk and ultimately reduce the positive rate of PTSD among this cohort. Additionally, physical health is an important factor causing PTSD among emergency nurses. Front-line workers in emergency medicine commonly have certain underlying diseases or health conditions, such as palpitations, chest tightness, hypertension, and lumbar disc protrusion. The discomfort caused by diseases can worsen the anxiety or depression of emergency nurses. Furthermore, due to the nature of emergency work, 83.57% of emergency nurses often work while ill. Therefore, studying the correlation between the physical condition of emergency nurses and their work positions is also a research direction that could help reduce the incidence of PTSD among emergency nurses in the future.

## 5. Conclusion

Based on the survey and research results, it is evident that emergency nurses have a high level of PTSD, with a positive rate of 26.04%. Several factors contribute to an increased probability of PTSD, and the psychological well-being of emergency nurses, needs to be addressed urgently. Future research can build upon the findings of this study to have a better understanding of the work, life, and psychological well-being of emergency nurses. Targeted interventions can be developed to reduce the occurrence of post-traumatic stress by dynamically regulating their working hours, improving their income level, and enhancing the nurse protection system. Furthermore, correlation studies related to post-traumatic growth can be conducted to further validate the effectiveness of these interventions. It is essential to acknowledge the limitations of this study. The sample size was relatively small, and the study was confined to the Jiangsu region of China, which may limit its generalizability and introduce potential bias in the results. Therefore, future studies should consider expanding the scope of the survey and conduct multicenter research to gain a broader understanding of the psychological status of emergency nurses, particularly during times when the incidence of emergency traumatic events is high globally.

## Data availability statement

The original contributions presented in the study are included in the article/supplementary material, further inquiries can be directed to the corresponding author.

## Ethics statement

The studies involving humans were approved by Ethics Committee of The Second Affiliated Hospital of Nantong University. The studies were conducted in accordance with the local legislation and institutional requirements. The participants provided their written informed consent to participate in this study.

## Author contributions

Y-FQ and YL conceived the idea and conceptualized the study. LW and QL collected the data. Y-FQ, LW, R-QS, and QL analyzed the data. YL obtained the financing and reviewed the manuscript. Y-FQ and R-QS drafted the manuscript. All authors contributed to the article and approved the submitted version.

## Funding

Jiangsu Hospital Association Hospital Management Innovation special research topic: investigation and intervention research of emergency nurses in public disaster events grant number: JSYGY-3-2021-JZ26.

## Conflict of interest

The authors declare that the research was conducted in the absence of any commercial or financial relationships that could be construed as a potential conflict of interest.

## Publisher’s note

All claims expressed in this article are solely those of the authors and do not necessarily represent those of their affiliated organizations, or those of the publisher, the editors and the reviewers. Any product that may be evaluated in this article, or claim that may be made by its manufacturer, is not guaranteed or endorsed by the publisher.
